# Factors associated with a late visit to dentists by children: A cross-sectional community-based study in Saudi Arabia

**DOI:** 10.1016/j.jtumed.2021.02.005

**Published:** 2021-03-11

**Authors:** Marwah Afeef, Nooralhuda Felemban, Noha Alhazmi, Zuhair S. Natto

**Affiliations:** a Study & Research Department, King Fahad Hospital, Jeddah, KSA; b Department of Oral & Preventive Medicine, King Fahad Hospital, Jeddah, KSA; c Department of Dental Public Health, Faculty of Dentistry, King Abdulaziz University, Jeddah, KSA

**Keywords:** مقدمي الرعاية, أول زيارة لطبيب الأسنان, مجتمع, أطفال, جدة, Cross-sectional, Community, First dental visit, Caregivers, Dependents, KSA

## Abstract

**Objectives:**

This cross-sectional study aims to determine children's age at their first visit to dentists and factors associated with these visits.

**Methods:**

This cross-sectional community survey-based study was conducted in 2019 during the events of the 10th Gulf Oral Health Week in Jeddah, Saudi Arabia. All participants including visitors and dentists, with current or previous experience in caring for children aged six months to ten years, provided their consents for the study.

**Results:**

Among the visitors, 348 participated in the survey. Most children, aged three to ten years, first visited the dentist with complaints of pain and dental cavities. The risk of a late visit to the dentist increased (OR: 2.28; CI 95%: 1.01–5.14) among caregivers who did not help their children brush their teeth. Using the Internet for accessing health information negatively impacted the visits (OR: 27.00; CI 95% 1.26–57.35). While employed mothers took their children to the dentist at an earlier age (OR: 2.284; CI 95% 1.08–4.79), early visits were mostly missed by mothers with smaller families (OR: 0.043; CI 95% 0.48–0.98).

**Conclusion:**

The results of our study show that the caregiver's attitude, source of health information, employment, age, and number of children are risk factors associated with late visits to dentists.

## الملخص

**أهداف البحث:**

تهدف هذه الدراسة المقطعية إلى تحديد عمر الأطفال أثناء الزيارة الأولى لطبيب الأسنان والعوامل المرتبطة بزيارتهم.

**طرق البحث:**

دراسة مجتمعية مقطعية تستند إلى الدراسة التي أجريت خلال فعاليات أسبوع الخليج العاشر لصحة الفم في مدينة جدة بالمملكة العربية السعودية ٢٠١٩. وشملت مقدمي الرعاية والزائرين الذين يرعون أو لديهم خبرة مع الأطفال الذين أعمارهم من ستة شهور إلى ١٠ سنوات والذين وافقوا على المشاركة في الدراسة الاستقصائية.

**النتائج:**

وافق ٣٤٨ زائرا على المشاركة واستكملوا الاستمارات، وقد زار معظم الأطفال طبيب الأسنان في سن ٣ إلى ١٠ سنوات. وكان أكثر أسباب الزيارة شيوعا هي الآلام والتجاويف، وازداد خطر زيارة أطباء الأسنان في سن متأخرة حيث معامل نسبة الأرجحية ٢.٢٨ بين مقدمي الرعاية الذين لا يساعدون في تنظيف أسنان أطفالهم، وقد أثر استخدام الإنترنت للمعلومات الصحية سلبا على الزيارات، وتأخذ الأمهات العاملات أطفالهن إلى طبيب الأسنان في سن مبكرة مقارنة بغير العاملات. كما لم تأخذ الأمهات اللاتي لديهن عدد قليل في سن متأخرة أطفالهم غالبا لزيارة الطبيب في سن مبكرة.

**الاستنتاجات:**

إن سلوك مقدمي الرعاية، ومصدر المعلومات الصحية، وحالتهم العملية، والعمر، وعدد الأطفال الذين تحت رعايتهم من العوامل المهمة والتي قد تؤدي إلى تأخر زيارات الأسنان.

## Introduction

Oral health is generally considered less important than general health. However, children should first visit their dentist as early as the eruption of their first tooth and no later than their first birthday. This is essential for avoiding early dental caries; facilitating the detection of early caries lesions; and helping caregivers in accessing the necessary oral health information to maintain their dependent's oral health and wellbeing.^1^^,^^2^ In KSA, unlike vaccination, no policies and practices govern children's first dental visit, leaving caregivers to decide on their dependents' first visit. Studies conducted in multiple cities in KSA have revealed a high prevalence of early childhood caries (ECC) among children aged 6 and younger**.**^3^, ^4^, ^5^, ^6^ For instance, the prevalence of ECC has been documented at 92% and 70–76% among children in Tabuk and Jeddah, respectively, with similar results produced in Al Karj and Riyadh.^(4)^ Caregivers in KSA tend to assume that age 3–6 is the optimal time for a child's first dental visit, or when they complain of pain.^2^^,^^7^ Early dental caries are a proven risk factor for caries in permanent dentition.^8^ Thus, to prevent dental caries, the importance of children first visiting the dentist at an age of below one year cannot be over-emphasized.

Few studies conducted in KSA investigated the age at which Saudi Arabian children first visited the dentist and the factors associated with these visits.^2^^,^^7^^,^^9^^,^^10^ For example, parents attending a dental clinic at King Saud University in Riyadh city reported a lack of knowledge about the optimal time to arrange their child's first dental visit; 62% of the parents were male.^10^ Moreover, Most children first visited the dentist at the age of 3–5 years.^9^ In Abha, located in the mountainous southern region of KSA, a convenient sample of caregivers attending dental clinics for the first time was included in the study. More than 60% of the children were aged 3–9 years when they first visited the dentist.^7^ These examples indicate a widespread lack of knowledge about the best age for children to visit the dentist for the first time. However, it is essential to recognize the factors contributing to this lack of knowledge, as opinions and attitudes are not formed in isolation.

Promoting the utilization of dental services for regular check-ups and preventive dental procedures is crucial for maintaining oral health.^11^ Since 2002, efforts to promote oral health have failed to show improvements due to insufficient knowledge about the social factors behind caregivers’ attitude towards early dental visits. In 1980, a new approach to disease causation, namely the socio-environmental approach, was introduced. This approach recognizes the impact on health outcomes of multiple social factors, which are referred to as the social determinants of health.^2^^,^^3^^,^^5^^,^^12^

In the city of Jeddah, efforts to contain the prevalence of caries have not been based on evidence for many years. There have been no previous studies in Jeddah that determine the age at which children visit the dentist for the first time, and little is known about caregivers’ attitudes and levels of oral care knowledge. Equally essential is breaking the cycle by asserting the factors associated with the first dental visits of children in Jeddah.

Thus, this study aims to examine caregivers in the community to determine the social factors associated with their dependents' dental visits; predict what factors influence the children's first visit to the dentist, to ensure that these factors are targeted in future oral health promotion programs; finally, recommend essential steps to decision makers for promoting early dental visits, attenuating the prevalence of ECC, and improving the oral health outcomes of children in KSA.

## Materials and Methods

### Study population

A cross-sectional community-based study has been conducted. The inclusion criteria were any caregivers residing in Jeddah who have currently or had previously cared for children aged 10 years and younger (6 months–10 years), who were attending the 10th Gulf Oral Health Week events that took place in community centres within Jeddah city, including the Jeddah seafront area. Convenience sampling was applied solely in Jeddah, and only those caregivers who had provided their consent to participate in the study were included. Caregivers who did not meet our inclusion criteria of caring, or who refused to participate, were consequently excluded from the study.

### Data collection

The study was based on data analysis from the survey titled ‘At what age do children of KSA first visit the dentist?’ This survey was conducted during the 10th Gulf Oral Health Week in Jeddah, KSA, in 2019. Visitors who provided their consent to participate in the survey were requested to fill out the questionnaire in Arabic.

The questionnaire was adapted from previous studies assessing caregivers' oral healthcare activities and practices^13^ and was translated and piloted for content and face validity. Three experts in paediatric dentistry evaluated each question's relevance, clarity, simplicity, and ambiguity. Face validity was then determined based on the feedback of ten caregivers whose first language was English, who were invited to read it, offer their opinions, and state whether they had found it difficult to understand or answer any of the questions. A native Arabic speaker translated the validated English questionnaire into Arabic with the required modifications. A native English speaker then re-translated the questionnaire into English, after which the versions in both languages were compared to confirm that they matched.

The questionnaire consisted of three sections and eighteen questions. The first section collected data on the caregiver's oral health attitude and knowledge; the second on the previous dental and general health experience; and the third on the caregiver's social demographics and characteristics. To help understand the socioeconomic factors of caregivers, we used the education level and employment status to avoid embarrassing caregivers or making them feel uncomfortable by asking them about their income.

### Sample size/power calculations

Sample size was calculated based on the estimated prevalence of delayed visit at 30% and 95% confidence interval using the G∗power program (version 3.1.9, Faul F, Erdfelder E, Lang A-G, & Buchner A, Germany). Therefore, 323 children were needed to achieve the desired estimated precision of 5%. The final sample size was suggested to be 350 in case of missing data.

### Data analysis

Data were processed using the software Statistical Package for Social Science (SPSS) version 25 (IBM Corp., Armonk, N.Y., USA). Descriptive statistics were used to describe all independent variables. Association between the dependent variable of the children's age at their first visit and the independent ones (demographics, oral health knowledge, and attitude) was tested using multinomial regression. *P* value of <0.05 was considered statistically significant. Children's age at their first visit was recoded into (early visit age from 6m to 2y, late visit age from 3y to 10y, and no visits).

## Results

### Study sample characteristics

A total of 348 questionnaires have been completed and analysed. Table 1 presents the characteristics of the participating caregivers, most of whom are female (91.7%) and the citizens of KSA (76.7%). In terms of age, most of our participants fall between the age groups of 25–34 (36.8%) and 35–44 (42.2%). Of the participants, (67.5%) are highly educated, having completed a bachelor's degree and above. In addition, only (39.1%) of the participating caregivers are employed. Most of our participants live in the northern region of Jeddah (39.2%).

**Table 1:** The characteristics of the participating caregivers.

Caregiver's Characteristics	n	%
*Gender*		
Female	319	91.7
Male	29	8.3
*Citizenship*		
Citizen	267	76.7
Resident	81	23.3
*Age group*		
24 & under	24	6.9
25–34	128	36.8
35–44	147	42.2
45–54	39	11.2
55 & older	10	2.9
*Relation to the child*		
Parent	314	90.2
Sibling	14	4.0
Grandparents	20	5.7
*Level of education*		
Bachelor & above	235	67.5
High school	81	23.3
Intermediate	17	4.9
Elementary	11	3.2
Others (illiterate)	4	1.1
*Employment*		
No	212	60.9
Yes	136	39.1
*Location*		
North Jeddah	127	39.2
South Jeddah	44	13.6
East Jeddah	20	6.2
Middle Jeddah	73	22.5
Out of Jeddah	60	17.2

### Oral health attitude and knowledge

**Figure 1:**
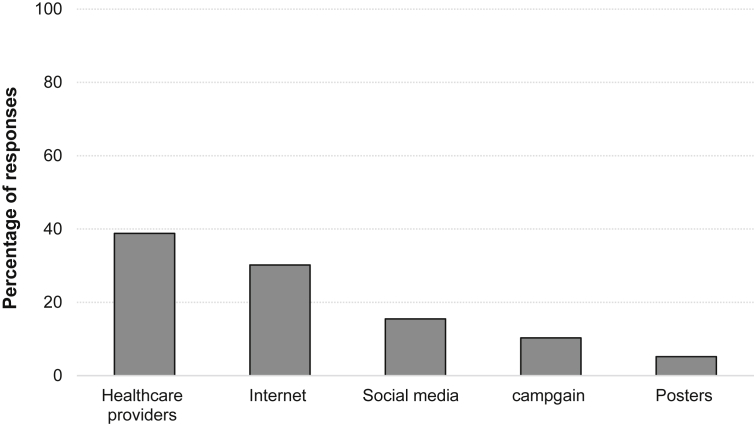
Caregivers' source of oral health information.

Caregivers' oral health attitude and knowledge are illustrated in Table 2. More than half of the caregivers (67.8%) report that they brush their dependents' teeth, and nearly half of the caregivers rate their dependents oral health as fair (52.3%). The two key sources of health information that most of the caregivers rely on are healthcare providers (38.8%) and the Internet (30.2%) (Figure 1). Almost all participants report that their dependents are updated with their vaccinations (96.3%). Contrarily, only (12.9%) of them report that their dependents visited the dentist at an early age (6m to 2y), while (23.6%) report that they never visited the dentist. More than half of the caregivers (62.2%) rate their dependents' behaviour at first visit as positive. When asked about the reason for their dependents’ first visit, (37.1%) report cavities to be the reason.

**Table 2:** Caregivers oral health attitude and knowledge.

Items	n	%
*Brushing your dependent's teeth?*		
Yes	236	67.8
No	112	32.2
*Describing dependent's OH*		
Excellent	136	39.1
Fair	182	52.3
Poor	30	8.6
*Your source of oral health information?*		
Social media	54	15.5
Healthcare provider	135	38.8
Campaign	36	10.3
Internet	105	30.2
Posters	18	5.2
*First visit to the dentists?*		
At early age 6m-2y	45	12.9
At late age 3y-10y	221	63.5
Never	82	23.6
*Updated with vaccine*		
Yes	335	96.3
No	13	3.7
*Reason for first visit?*		
Pain	64	24.2
Check up	70	26.5
Cavities	98	37.1
Referrals	2	0.8
Fluoride	19	7.2
Others (extractions of baby teeth)	11	4.2
*Dependent's behaviour?*		
Negative	99	37.8
Positive	163	62.2

### Factors associated with first visit

Some factors associated with the caregivers' attitudes towards their dependents' first visit to the dentist are outlined in Table 3. For those caregivers who state that they do not brush their dependents' teeth compared with those who state that they do, the probability of visiting the dentist at a late age compared with an early age is expected to increase by (OR: 2.28; CI 95%: 1.01–5.14), given the other variables in the model are held constant. For those caregivers who state that they do not brush their dependents' teeth compared with those who state that they do, the probability of never visiting the dentist compared with visiting at an early age is expected to increase by (OR: 2.81; CI 95%: 1.16–6.81) given the other variables in the model are held constant. Thus, the act of brushing the dependents' teeth positively impacts the caregiver's attitude about their dependents' first visit to the dentist.

**Table 3:** Factors associated with first visit to the dentist.

Independent variables	Late visit∗		Never visited∗	
	OR	[95% CI]	OR	[95% CI]
*Gender*				
Female	1.00	[Reference]	1.00	[Reference]
Male	0.57	[0.21–1.54]	0.42	[0.12–1.47]
*Citizenship*				
Citizen	1.00	[Reference]	1.00	[Reference]
Resident	1.11	[0.50–2.46]	1.65	[0.69–3.95]
*Age group*				
24 & under	7.00	[0.60–81.68]	27.00	[1.26–57.35]
25–34	2.05	[0.46–9.01]	6.00	[0.58–61.84]
35–44	3.15	[0.71–13.90]	5.62	[0.54–58.57]
45–54	1.85	[0.36–9.36]	2.57	[0.20–31.71]
55 & older	1.00	[Reference]	1.00	[Reference]
*Level of education*				
Bachelor & above	0.87	[0.44–1.75]	1.09	[0.49–2.40]
High school & under	1.00	[Reference]	1.00	[Reference]
*Employment*				
No	2.11	[1.10–4.05]	2.28	[1.08–4.79]
Yes	1.00	[Reference]	1.00	[Reference]
*Location*				
North	0.69	[0.27–1.76]	0.59	[0.20–1.74]
South	0.56	[0.16–1.92]	1.58	[0.43–5.75]
East	1.07	[0.19–5.89]	1.50	[0.23–9.44]
Middle	1.61	[0.50–5.18]	1.08	[0.28–4.08]
Out of Jeddah	1.00	[Reference]	1.00	[Reference]
*Brush dependent teeth*				
Yes	1.00	[Reference]	1.00	[Reference]
No	2.28	[1.01–5.14]	2.81	[1.16–6.81]
*Dependent OH*				
Excellent	0.60	[0.20–1.78]	2.93	[0.64–13.32]
Fair	2.00	[0.66–6.05]	3.88	[0.82–18.39]
Poor	1.00	[Reference]	1.00	[Reference]
*Source of OH info*				
Social media	1.03	[0.27–3.85]	1.63	[0.24–11.07]
Healthcare providers	1.66	[0.48–5.72]	2.21	[0.36–13.47]
Campaign	1.40	[0.31–6.23]	4.00	[0.53–29.80]
Internet	3.27	[0.80–13.41]	13.33	[1.99–89.31]
Posters	1.00	[Reference]	1.00	[Reference]
*Reason for 1*st *visit*				
Pain	1.75	[0.31–9.86]		
Check up	0.56	[0.11–2.81]		
Cavities	1.79	[0.34–9.41]		
Fluoride	0.16	[0.02–0.94]		
Extraction of baby teeth	1.00	[Reference]		
*Number of dependents*	1.26	[0.95–1.67]	0.68	[0.48–0.98]
*Relation to the dependent*				
Parents	1.52	[0.47–4.91]	2.53	[0.54–11.87]
Sibling	3.07	[0.29–31.98]	4.00	[0.26–60.32]
Grandparents	1.00	[Reference]	1.00	[Reference]
*Dependent behaviour*				
Positive	1.00	[Reference]	1.00	[Reference]
Negative	1.07	[0.54–2.10]		

∗ Compared with early visit.

The model of predicting an early visit to the dentist based on the caregiver's opinion about their dependents' oral health status is significant with a *P* value of <0.05. However, individually, none of the predictors are significant. For those caregivers who state that they rely more heavily on the Internet for oral health information compared with those who state that they rely on posters, the probability of never visiting the dentist compared with a visit at an early age is expected to increase by (OR: 13.33; CI 95% 1.99–89.31), given the other variables in the model are held constant. Internet utilization for health information therefore contributes negatively to the caregiver's attitude toward early dental visits.

For those caregivers who state that they visit the dentist for preventive measures, such as fluoride, compared with those who report that they visit the dentist to extract baby teeth, the probability of visiting the dentist at a late age compared with a visit at an early age is expected to decrease by (OR: 0.164; CI 95% 0.02–0.94), given the other variables in the model are held constant. Thus, regular visits to the dentist for receiving fluoride promote early visits, thereby preventing extraction visits for those children.

For those caregivers who are young (24 years and under) compared with those who are 55 years and older, the probability of never visiting the dentist compared with a visit at an early age is expected to increase by (OR: 27.00; CI 95% 1.26–57.35), given the other variables in the model are held constant. Being a young mother could negatively impact the child's first visit to the dentist. For those caregivers who are not employed compared with those who are, the probability of never visiting the dentist compared with a visit at an early age is expected to increase by (OR: 2.284; CI 95% 1.08–4.79), given the other variables in the model are held constant. Employed mothers confirm that they take their children to the dentist at an early age. Surprisingly, if the number of children were to increase by one, the probability of never visiting the dentist to an early visit is expected to decrease by a factor of (OR: 0.68; CI 95% 0.48–0.98), given the other variables in the model are held constant. Thus, those who have a smaller number of children are more likely to never visit the dentist.

## Discussion

A dental visit in children's early years is crucial for them for ensuring that they are exposed to professional and preventive dental services as early as possible.^11^ This study shows that most of the children had visited the dentist for the first time when they were between the ages of 3 and 10 years, and a small number of children had visited when they were 2 years old. In addition, the most common reason given for their first visit is reported to be pain and cavities. Our results agree with multiple national and international studies investigating the age of children at their first dental visit. In Riyadh, the capital of KSA, 3–5 years is revealed as the age at which most children have their first dental visit.^9^ Similar studies in Bulgaria and India have shown that a greater number of children visit the dentist for the first time at the age of 3–10.^14^^,^^15^ Furthermore, in the United States, multiple studies have agreed and confirmed that a small percentage of caregivers had taken their children to their first dental visit by the age of 2.^2^^,^^16^

Oral care should begin as early as the eruption of a child's first tooth. Multiple factors have been found to be significant in predicting their first visit to the dentist. For instance, caregivers' oral care attitude and knowledge were associated with their dependents' first visit to the dentist. Our results agree with a study confirming that caregivers' oral health knowledge and attitude significantly influence the oral health of their dependents.^16^ One of this study's objectives is to determine caregivers' oral health care attitude and knowledge to identify and amend any gaps, thereby helping to improve their dependents' oral health. This study shows that most of the participating caregivers are the mothers of the children in their care and are of childbearing age. Unsurprisingly, our findings agree with a considerable number of other studies that see mothers as the primary caregivers of their children, actively involved in the everyday tasks of caregiving.^17^^,^^18^ In addition, more than half of our participating caregivers are highly educated, with a bachelor's degree and above. However, more than half are housewives.

Almost all participants in the study report their dependents to be updated with their vaccinations, indicating that they view preventive general health measures as important and beneficial. In addition, most of the caregivers have satisfactory oral health knowledge and attitude. An adequate number of caregivers demonstrate a positive attitude toward oral care, valuing the act of helping their dependents brush their teeth. The act of brushing the dependents' teeth has been found to positively impact the caregiver's attitude toward their dependents' first visit to the dentist. Moreover, helping the child to brush their teeth exposes the caregivers to any changes in their oral health, leading them to take immediate action if necessary. When caregivers are asked to describe the health of their dependents' teeth, nearly half of them rate it as fair. The caregivers' perception of their dependents' oral health as good, fair, or poor has been found to have a weak influence on their attitude toward their dependents' early visits. Generally, caregivers do not perceive oral health as a health threat, which explains their attitude towards early preventive visits. Moreover, most of the children are apparently cooperative, as their behaviour at first visit is largely rated as positive.

The present study has found that a considerably low (38.8%) proportion of caregivers utilize healthcare providers as their key source of health information, and lower than the proportion (60% and above) reported in multiple studies assessing caregivers' knowledge and main source of oral health information.^19^, ^20^, ^21^ Visiting healthcare providers for health information is associated with costs and waiting time. However, the introduction of alternative health information channels, such as social media and the Internet, facilitates caregivers’ access to health information with minimal cost, time, and travel. The public has become highly skilled in using computers and the Internet. Another study confirms that participants who report long travel time for obtaining health information are more likely to access other communication channels to gain health information.^22^

Moreover, the results of this study confirm that the children of young mothers are at risk of never visiting the dentist compared with those of older mothers or caregivers.^23^^,^^24^ Additionally, the risk of unemployed caregivers and mothers never taking their dependents to the dentist compared with taking them at an early age is expected to increase. Surprisingly, if the number of children were to increase by one, the risk of never visiting the dentist is expected to decrease. Therefore, mothers or caregivers with a smaller number of children are more likely to never visit the dentist.^25^, ^26^, ^27^, ^28^, ^29^, ^30^, ^31^

The present findings have some implications. There is certainly a delay on the first visit to the dentist. Current strategies to target early childhood caries must consider the factors associated with delayed dental visits. Further contact with policy makers may help to manipulate the factors associated with this delay and promote early dental visits. Tailored methods are mandatory for appropriately educating caregivers in recognizing and acting on changes to their dependents’ oral health. Our findings may encourage the development of oral health promotion programs targeting new mothers and parents of pre-school children.

This study has some limitations that may impact the results. First, the data collected are self-reported and may therefore contain bias, as respondents may have been too embarrassed to disclose private details. Thus, bias and social desirability may affect the results. Additionally, oral screening should have been included, as this would have provided insight into the dependents’ oral care and health status.

## Conclusion

This is the first community study in Jeddah city determining the age and factors associated with children's first visit to the dentist. Caregivers' attitude, source of health information, employment status, age, and the number of children that they care for are found to be major risk factors for dental visits. Establishing practices to promote early dental visits not later than the age of 1 is crucial. Finally, raising caregivers' awareness about the age at which their children must first visit the dentist, and about these visits' significance, may be the first step in promoting the prevention of early childhood caries.

## Recommendations

Oral health initiatives targeting new caregivers will help promote their dependents' oral health; the provision of oral prophylaxis and workshops on appropriate oral care would empower caregivers to support and improve their children's oral health. Moreover, healthcare providers must deliver concise and clear professional oral health messages advising caregivers to take their dependents for a visit to the dentist no later than the age of 1. Additionally, the caregivers of preschool-age children must receive sufficient attention, with preventive oral health initiatives — such as fluoride varnish campaigns — targeting this age group.

## Source of funding

This research did not receive any specific grant from funding agencies in the public, commercial, or not-for-profit sectors.

## Conflict of interest

The authors have no conflicts of interest to declare.

## Ethical approval

The study protocol was registered with the Research and Studies Department- Jeddah Health Affairs. The IRB registration number with KACST, KSA: H-02-J-002/; Research number: 01038. All participants gave written informed consent before participating in this study. Ethical Approval was done by ethical committee of Jinnah Post Graduate Medical Centre (No. F 5-89/2015/GENL/599/JPMC dated 1st February 2015).

## Authors contributions

MA, the first author, contributed to this study by helping in the conception, design, defining intellectual content, literature search, data analysis, manuscript preparation, editing, and review, and will be the manuscript guarantor. The second author, NF, contributed to this study by helping in the conception, design, defining intellectual content, literature search, and manuscript preparation, editing, and review. The third and the fourth author, NA and ZN, contributed to this study by helping in the conception, design, defining intellectual content, literature search, and manuscript preparation, editing, and review. All authors have critically reviewed and approved the manuscript's final draft and are responsible for its content and similarity index.
